# Most Common Publication Types of Neuroimaging Literature: Papers With High Levels of Evidence Are on the Rise

**DOI:** 10.3389/fnhum.2020.00136

**Published:** 2020-04-28

**Authors:** Andy Wai Kan Yeung

**Affiliations:** Oral and Maxillofacial Radiology, Applied Oral Sciences and Community Dental Care, Faculty of Dentistry, The University of Hong Kong, Hong Kong, China

**Keywords:** publication type, neuroimaging, diagnostics, bibliometric, citation distribution, uncitedness

## Abstract

**Objective:** This study evaluated the bibliometric data of the most common publication types of the neuroimaging literature.

**Methods:** PubMed was searched to identify all published papers with “neuroimaging” as their MeSH Major Topics, and they were further searched by the following publication types: case report, clinical trial, comparative study, editorial, evaluation study, guideline, meta-analysis, multicenter study, randomized controlled trial, review, technical report, and validation study. The proportion of papers belonging to each publication type published in neuroimaging journals was calculated. Year-adjusted mean citation counts for each publication type were computed using data from Web of Science. Publication trend and its correlation with citation performance were assessed.

**Results:** Review and comparative study were the most common publication types. Publication types with the highest proportion in neuroimaging journals were guideline, validation study, and technical reports. Since the year 2000, multicenter study, review, and meta-analysis showed the strongest linear increase in annual publication count. These publication types also had the highest year-adjusted citation counts (4.7–10.0). Publication types with the lowest year-adjusted citation counts were editorial and case report (0.5–1.0). It was estimated that 12.5% of the publications labeled as case reports were incorrectly labeled.

**Conclusions:** Neuroimaging literature has been expanding with papers of higher levels of evidence, such as meta-analyses, multicenter studies, and randomized controlled trials.

## Introduction

Neuroimaging can provide useful diagnostic images and experimental findings that inform and support evidence-based clinical practice. For example, diffusion-weighted imaging (DWI) is considered as a useful modality to diagnose patients with acute ischemic stroke (Schellinger et al., 2010). Magnetic resonance images are also useful for many neurologic conditions such as diagnosing posterior reversible encephalopathy syndrome (Lamy et al., 2004) and detecting white matter alterations in early stages of schizophrenia (Samartzis et al., 2014). Also, positron-emission tomography (PET) and single-photon emission computed tomography (SPECT) can provide useful diagnostic biomarkers for Alzheimer's disease (Mueller et al., 2005). Clinicians, scientists, and policy-makers rely on the scientific evidences reported by peer-reviewed literature to determine if certain management methods and strategies should be recommended or not. Not only journals are judged with their credibility (Manca et al., 2017), the journal publications themselves are also associated with different levels of evidence—from systematic reviews and meta-analyses of randomized trials to case series and mechanism-based reasoning (Winkelmann et al., 2013).

The distribution of papers in these publication types was assessed in the radiology field (Rosenkrantz et al., 2016). Surely, the various publication types differed in not only the publication count but also the number of downloads and citations (Moed and Halevi, 2016; Rosenkrantz et al., 2016). Though numerous surveys have been conducted for the neuroimaging or neuroscience literature to assess its popular topics (Yeung et al., 2017b; Yeung, 2018b), geographic distribution of institutions and countries that contributed to highly cited papers (Bornmann et al., 2011), text readability (Yeung et al., 2018), prevalent statistical thresholds (Yeung, 2018c), and even editorial stance toward replication studies (Yeung, 2017), no study has reported the distribution and citation of publication types. The current study, as a conceptual replication of Rosenkrantz et al. (2016), assessed the publication types in neuroimaging literature, their correlations with citation performance and distribution, and the temporal trends of publications.

## Methods

Relevant publications were identified *via* PubMed searches conducted in July 2019. Only publications with “neuroimaging” being one of their MeSH Major Topics were included. Moreover, each search was limited by a specific “Publication Type” assigned by Medline staff to the publications indexed in PubMed. Following the search method by Rosenkrantz et al. (2016), 12 “Publication Types” were considered: case report, clinical trial, comparative study, editorial, evaluation study, guideline, meta-analysis, multicenter study, randomized controlled trial, review, technical report, and validation study. The total number of publications for each publication type was recorded, and their PMIDs were searched *via* the Web of Science (WoS) Core Collection database. As WoS but not PubMed records citation data, only publications that were indexed in WoS were further evaluated in the current study. The Journal Citation Reports (JCR) assigns journals to different categories, one of which being “Neuroimaging.” The proportion of publications of each publication type published within JCR “Neuroimaging” journals was computed. The three most common journals for each publication type were identified.

To reveal potential recent linear publication trends, the annual publication counts for each publication type since year 2000 were recorded and entered into linear regression models. Besides, Pearson correlation test was used to assess if, across the publication types, year-adjusted mean citation count was associated with the proportion of publications in neuroimaging journals or the proportion of uncited publications. To give a better picture of citation distribution for each publication type, the proportion of publications with more than the year-adjusted mean citation count was recorded. Statistical assessment was performed in SPSS 25.0 (IBM, New York, USA). Tests with *P* < 0.05 were considered statistically significant.

## Results

The proportion of WoS coverage for PubMed-indexed publications ranged from 81.1% (guideline) to 99.3% (meta-analysis). The smallest publication type was technical report (*n* = 16 in WoS), whereas the largest type was review (*n* = 4,862) (Table 1). From the data, over one-third of neuroimaging guidelines were published in neuroimaging journals, but only one-sixth of neuroimaging reviews were so. *NeuroImage* seemed to be a popular journal for most of the publication types, whereas other journals had specific niches, such as *American Journal of Neuroradiology* for guidelines, clinical trials, and multicenter studies, *IEEE Transactions on Biomedical Engineering* for validation and evaluation studies, and *Stroke* for multicenter studies and clinical trials.

**Table 1:** Proportion of neuroimaging papers of various publication types published in neuroimaging journals and the three most common journals publishing them.

**Publication type**	**% in neuroimaging journals**	**Three most common journals (%)**
Guideline (*n* = 30)	36.7	American Journal of Neuroradiology (16.7)
		Journal of Vascular and Interventional Radiology (16.7)
		Journal of Neuroimaging (13.3)
Validation study (*n* = 440)	26.4	NeuroImage (11.1)
		IEEE Transactions on Biomedical Engineering (6.6)
		Human Brain Mapping (5.7)
Technical report (*n* = 16)	25.0	Human Brain Mapping (18.8)
		Acta Neurochirurgica (12.5)
		World Neurosurgery (12.5)
Editorial (*n* = 630)	23.3	American Journal of Neuroradiology (12.1)
		Neurology (4.3)
		Clinical Neuroradiology (3.7)
Meta-analysis (*n* = 432)	23.1	Human Brain Mapping (8.1)
		NeuroImage (7.9)
		Neuroscience and Biobehavioral Reviews (7.9)
Multicenter study (*n* = 366)	22.1	Stroke (9.8)
		Human Brain Mapping (6.0)
		American Journal of Neuroradiology (4.4)
Randomized controlled trial (*n* = 641)	19.5	NeuroImage (7.6)
		Journal of Neuroscience (5.5)
		Neuroradiology (3.3)
Case report (*n* = 2,628)	19.1	American Journal of Neuroradiology (7.2)
		Neuroradiology (6.2)
		Journal of Neurosurgery (4.6)
Evaluation study (*n* = 1,022)	18.3	NeuroImage (9.8)
		IEEE Transactions on Biomedical Engineering (6.0)
		Magnetic Resonance in Medicine (3.6)
Comparative study (*n* = 4,686)	17.8	American Journal of Neuroradiology (4.3)
		NeuroImage (4.2)
		Human Brain Mapping (3.1)
Clinical trial (*n* = 2,035)	17.8	NeuroImage (5.6)
		Stroke (5.3)
		American Journal of Neuroradiology (4.2)
Review (*n* = 4,862)	16.7	NeuroImage (6.8)
		Neuroimaging Clinics of North America (3.2)
		Neuroscience and Biobehavioral Reviews (2.8)

*Neuroimaging journals refer to journals classified by Journal Citation Reports (Clarivate Analytics) as in the Neuroimaging category*.

Since year 2000, seven publication types showed significant linear increase, namely, randomized controlled trial, meta-analysis, multicenter study, technical report, review, case report, and editorial (Table 2). Multicenter studies, reviews, and meta-analyses showed the strongest linear increase in annual publication count. These publication types also had the highest year-adjusted citation counts (4.7–10.0), whereas case reports and editorials were scarcely cited (0.5–1.0) (Supplementary Table 1). Regarding the citation distribution, clinical trials, guidelines, and randomized controlled trials seemed to have a more even citation distribution, with ~30% of papers having their year-adjusted citation count above the average. In particular, all guidelines received at least one citation. Meanwhile, only 18.8% of technical reports had an above-average year-adjusted citation count and 12.5% had zero citation, implying that few technical reports were highly cited and skewed the citation distribution. Finally, across the publication types, year-adjusted mean citation count did not correlate with proportion in neuroimaging journals (*r* = 0.021, *p* = 0.947) and proportion of uncited publications (*r* = −0.502, *p* = 0.096).

**Table 2:** Temporal trends in the publication count of neuroimaging papers of various publication types.

**Publication Years**	**Review**	**Comparative study**	**Clinical trial**	**Case report**	**Evaluation study**	**Editorial**	**Randomized controlled trial**	**Validation study**	**Meta-analysis**	**Multicenter study**	**Guideline**	**Technical report**
2000	66	89	49	63	8	11	3	2	3	3	1	0
2001	50	75	57	49	18	11	15	8	3	6	1	0
2002	41	108	65	60	23	10	6	6	2	2	0	0
2003	82	239	90	74	75	12	7	49	2	2	0	0
2004	104	313	152	78	46	7	15	42	0	4	2	0
2005	103	457	145	57	75	12	14	37	2	9	2	0
2006	138	460	118	65	56	13	22	10	3	8	0	0
2007	162	166	58	73	72	15	10	16	5	8	1	1
2008	124	177	72	56	55	26	21	24	9	9	1	0
2009	131	112	50	70	72	31	15	13	10	11	4	3
2010	161	163	51	97	49	29	20	17	12	14	1	0
2011	254	190	89	108	37	39	43	31	19	16	1	2
2012	489	202	107	143	29	53	53	20	58	28	3	1
2013	376	203	124	126	46	75	60	32	46	28	1	0
2014	482	206	139	115	56	60	65	38	53	34	0	2
2015	425	155	89	118	70	55	35	23	41	36	4	0
2016	381	149	84	110	65	49	42	25	57	43	3	2
2017	423	107	53	102	50	41	32	26	60	40	2	4
2018	358	79	59	46	18	36	29	18	40	38	1	1
Since 2000	4,350	3,650	1,651	1,610	920	585	507	437	425	339	28	16
*R*^2^	0.790	0.043	0.000	0.332	0.032	0.664	0.540	0.046	0.759	0.885	0.141	0.324
*P* value	<0.001^*^	0.392	0.959	0.010^*^	0.460	<0.001^*^	<0.001^*^	0.379	<0.001^*^	<0.001^*^	0.114	0.011^*^

*Linear regression was conducted for each publication type to assess if the annual publication count showed a significant linear increase or decrease*.

* *P < 0.05*.

It was counterintuitive to see the growth in the annual publication count of case reports, given that most journals do not accept them nowadays unless they have exceptional clinical merit. Therefore, a two-part *post hoc* analysis was performed. First, upon a closer examination, the case reports were actually published in 93 journals during the 1980s, 134 during the 1990s, 189 during the 2000s, and 268 during the 2010s. It is reasonable to deduce that the newer journals commenced publication served as the venue for some of the newer case reports, given that the numbers of case reports and journals publishing them formed an apparently linear relationship over the last four decades (Supplementary Figure 1). Meanwhile, the definition of case reports by PubMed/Medline is “clinical presentations that may be followed by evaluative studies that eventually lead to a diagnosis” (https://www.nlm.nih.gov/mesh/pubtypes.html), which is arguably a bit vague. On the other hand, the National Cancer Institute (NCI) defined case report as “a detailed report of the diagnosis, treatment, and follow-up of an individual patient. Case reports also contain some demographic information about the patient (for example, age, gender, ethnic origin)” (https://www.cancer.gov/publications/dictionaries/cancer-terms/def/case-report). As Table 2 illustrates a steady growth of case reports since 2000, the second part of the *post hoc* analysis was a manual screening to determine if the labeled “case reports” were labeled correctly. The definition by NCI was referenced, with a modification that multiple patients were allowed. Five percent of the 1,610 case reports marked in Table 2 were assessed (*n* = 80). A random sequence was generated from www.random.org, and the corresponding items were picked from the list of 1,610 case reports sorted by date (newest to oldest). The author determined that 10 of the 80 publications were not case reports, meaning a tagging accuracy of 87.5% for case reports, and that the number of case reports was not inflated.

## Discussion

There were huge variations in publication and citation data among various publication types. Consistent to previous reports (Chew and Relyea-Chew, 1988; Rosenkrantz et al., 2016), case reports that belong to lower levels of evidence had fewer citations relative to their counterparts with higher levels of evidence, such as meta-analyses and randomized controlled trials. However, unlike previous reports, the citation data did not correlate with the proportion of publications in specialized (neuroimaging) journals (Rosenkrantz et al., 2016) and proportion of uncited publications (Yeung, 2019b). Though a direct comparison may not be possible, the current study showed that reviews had 1.6 times more year-adjusted citations than guidelines, which seemed to be the reverse of the situation in radiology, where the latter had two times more 2-year citations than the former (Rosenkrantz et al., 2016).

Regarding citations, readers should be aware of the fact that not all citations are the same. The underlying citing behavior is a complicated meta-theoretical matter (Leydesdorff, 1998) that may not be solely acknowledging the intellectual and cognitive influences of preceding work, but including eight major types: affirmational, assumptive, conceptual, contrastive, methodological, negational, perfunctory, and persuasive (Bornmann and Daniel, 2008). Apart from the citation context and polarity, the semantics and linguistic patterns in citations as well as the citation locations within the text also account for the citing behavior (Tahamtan and Bornmann, 2019). It implied that citation count is an overall value that has multiple facets in various proportions. Metrics were developed to transform citation count in different contexts, for instance, the source normalized impact per paper (SNIP) at the journal level (Moed, 2010), the relative citation ratio at the article level (Hutchins et al., 2016), and so on. Uncitedness may relate to lower impact journals (Garfield, 1998b; Van Leeuwen and Moed, 2005), but many uncited papers would eventually receive citations after some time had lapsed (Garfield, 1998a). Besides, uncitedness tended to decrease in the digital age as the reliance on journal impact factor to attract citations for individual papers has been diminished (Lozano et al., 2012), the size of the publisher did not seem to affect the relative citation rate of papers (Larivière et al., 2015), and the number of references per paper increased (Wallace et al., 2009).

It is a common notion that review papers are more cited than original research papers, with a recent analysis, based on the publication types used by WoS database, suggesting that the former are generally cited three times more than the latter (Miranda and Garcia-Carpintero, 2018). In neuroimaging journals, the ratio is about 2.5–1 (Miranda and Garcia-Carpintero, 2018). However, the current results suggested that the whole picture behind this simple ratio could be much more complicated. First, review papers with meta-analyses were much more cited than pure reviews. Similarly, once the original research papers were further divided by numerous publication types, readers could recognize that multicenter studies and randomized controlled trials were much more cited than case reports, which constituted a large proportion of publications analyzed in the current study. At first, it might be counterintuitive to see the continued growth of case reports, given that most journals do not accept them unless they have exceptional clinical merit. As reported in the *Results*, one reason for the growing number of case reports is that there is a growing number of (new) journals publishing them. Also, many case reports do not merely report a case. Some of them are accompanied by a short review of the literature, and some of them reported many patients as a case series. The boundary between a case report and other publication types, such as a retrospective study without control groups and analytical statistics, can be quite vague. Moreover, a minority of publications could be wrongly labeled by PubMed/Medline as case reports, e.g., a prospective observational study with 4,568 patients, and a structured abstract with background, methods, results, and conclusion (Gupta et al., 2007). Perhaps both journal editorial boards and data tagging staff of bibliometric databases should consider how to better define and distinguish the coverage of “case reports.”

It is reassuring to see that randomized controlled trials and multicenter studies have been on a steady rise since the beginning of the 2000s. A previous report has pointed out that funding could be the main driver for the continual increase in publications (Lariviere et al., 2013). Surely, these research types represent higher levels of evidence relative to case reports and retrospective studies. Nonetheless, readers should be aware of other aspects of neuroimaging studies that may influence the quality of scientific evidence, namely, the sample size and statistical threshold. Small samples with uncorrected statistics might inflate the chance of having false positive results (Poldrack et al., 2017). A series of surveys of neuroimaging papers seemed to show that the statistical thresholds have been becoming more stringent, but the sample size is still quite small in general (Guo et al., 2014; Woo et al., 2014; Yeung, 2018c).

This study has several inherited limitations. To begin with, not all PubMed-indexed publications are tagged with labels such as “Publication Types” and “MeSH Major Topics,” which are assigned by Medline. Still, it was advocated that MeSH-based search strategy should be preferred, and programs were developed to facilitate the work (Lundberg et al., 2006; Leydesdorff et al., 2012; Leydesdorff and Opthof, 2013). In addition, a minority of the PubMed-indexed publications was not indexed by WoS and thus excluded from the current study. Therefore, the current analyzed literature set could not fully represent the entire neuroimaging literature. Moreover, results may be different if alternative databases such as Scopus would be used. Readers should also be aware of the large values of standard deviations listed for the year-adjusted citation count of the publication types in Supplementary Table 1, which might be reduced by transforming the data to a ratio of citation counts of each type to that of the most-cited type (Miranda and Garcia-Carpintero, 2018), i.e., meta-analysis. Meanwhile, the percentage of uncited publications could be biased/inflated if certain publication types were preferentially published in the recent years and thus had less time to receive citations. Future surveys should also consider applying the percentage of publications among different quartiles by journal's impact factor as a metric to evaluate citation performance besides citation count (Miranda and Garcia-Carpintero, 2019).

Given the large number of publications involved in the current study, the author was unable to manually screen every publication to determine the accuracy of publication type. However, previous studies demonstrated that the publication types could be assigned incorrectly, in the range of 1.9–29.3% (Donner, 2017; Yeung, 2019a). For instance, the current results showed that there were only 30 neuroimaging guidelines. The author was aware of a renowned guideline recently published by the neuroimaging meta-analysis community, entitled “Ten simple rules for neuroimaging meta-analysis” (Müller et al., 2018). One of its keywords was “guidelines.” Meanwhile, Medline labeled its “Publication Type” as a “review,” and “MeSH Major Topics” as “Guidelines as Topic,” “Meta-analysis as Topic,” and “Neuroimaging.” The author wished to highlight the confusion caused by the overlapping between “Publication Type” and “MeSH term” labeling in this example (Müller et al., 2018). In fact, the “Publication Type” tags by Medline were heavily relied on by routine searches and bibliometric analyses in health sciences (Mosa and Yoo, 2013; Ma et al., 2016). Ideally, the tagging rules should be easier and simpler, so that users are able to identify relevant body of literature more efficiently, without advanced or complicated search queries. Meanwhile, the current study also did not distinguish the imaging modalities used in the publications, though previous bibliometric studies reported that magnetic resonance imaging was the predominant imaging modality for the top 100 most cited neuroimaging papers as well as the whole neuroimaging literature (Kim et al., 2016; Yeung et al., 2017b,c).

This work surveyed the publication types of the neuroimaging literature. It added another perspective to how the literature shaped. Readers should also refer to other systematic reviews, bibliometric reports, and opinion articles to better grasp the overview of various aspects of the field. For instance, machine learning or pattern classification has been popular since the early 2010s, and it helps the field to develop into the direction of individualized biomarkers of diseases or functional brain states (Davatzikos, 2019). Regarding the most cited papers in the field, readers can refer to previous works which showed that neurological disorders and emotion/reward were recurring themes (Yeung et al., 2017a), human subjects were more common than animal models, and magnetic resonance imaging was more prevalent than positron emission tomography (Yeung, 2018a). Reproducibility has been an issue receiving attention. With the advancement in the statistical modeling and validations, the use of uncorrected statistics in neuroimaging literature dropped from 41% reported in 2012 (Carp, 2012) to around 4.4% near the end of the 2010s (Yeung, 2018c). For the publications on top of the level of evidence pyramid, i.e., meta-analyses, increasingly more stringent statistical thresholds were adopted, but the number of studies contained in the analyses did not significantly rise (Yeung et al., 2019).

In conclusion, the neuroimaging literature has been expanding with papers of higher levels of evidence, such as meta-analyses, multicenter studies, and randomized controlled trials, though case reports are still a large part of the literature. More neuroimaging guidelines and technical reports should be encouraged.

## Author Contributions

The author confirms being the sole contributor of this work and has approved it for publication.

## Conflict of Interest

The author declares that the research was conducted in the absence of any commercial or financial relationships that could be construed as a potential conflict of interest.

## Supplementary Material

Supplementary Figure 1 An apparent linear relationship between the number of case reports published in each decade and the corresponding number of journals publishing them.

Supplementary Table 1 Year-adjusted citation counts of various publication types of neuroimaging papers, in descending order.

## References

[B1] Bornmann L. Daniel H. D. (2008). What do citation counts measure? A review of studies on citing behavior. J. Doc. 64, 45–80. 10.1108/00220410810844150

[B2] Bornmann L. Leydesdorff L. Walch-Solimena C. Ettl C. (2011). Mapping excellence in the geography of science: an approach based on Scopus data. J. Informetr. 5, 537–546. 10.1016/j.joi.2011.05.005

[B3] Carp J. (2012). The secret lives of experiments: methods reporting in the fMRI literature. Neuroimage 63, 289–300. 10.1016/j.neuroimage.2012.07.004 22796459

[B4] Chew F. S. Relyea-Chew A. (1988). How research becomes knowledge in radiology: an analysis of citations to published papers. Am. J. Roentgenol. 150, 31–37. 10.2214/ajr.150.1.31 3257128

[B5] Davatzikos C. (2019). Machine learning in neuroimaging: progress and challenges. Neuroimage 197, 652–656. 10.1016/j.neuroimage.2018.10.003 30296563 PMC6499712

[B6] Donner P. (2017). Document type assignment accuracy in the journal citation index data of web of science. Scientometrics 113, 219–236. 10.1007/s11192-017-2483-y

[B7] Garfield E. (1998a). Long-term vs. short-term impact: Part II. cumulative impact factors. Scientist 12, 12–13.

[B8] Garfield E. (1998b). Untitled and anonymous editorials and other forms of provincialism. Scientist 12:8.

[B9] Guo Q. Parlar M. Truong W. Hall G. Thabane L. Mckinnon M.. (2014). The reporting of observational clinical functional magnetic resonance imaging studies: a systematic review. PLoS ONE 9:e94412. 10.1371/journal.pone.0094412 24755843 PMC3995931

[B10] Gupta R. Vora N. Thomas A. Crammond D. Roth R. Jovin T.. (2007). Symptomatic cerebral air embolism during neuro-angiographic procedures: incidence and problem avoidance. Neurocrit. Care 7, 241–246. 10.1007/s12028-007-0041-9 17805494

[B11] Hutchins B. I. Yuan X. Anderson J. M. Santangelo G. M. (2016). Relative citation ratio (RCR): a new metric that uses citation rates to measure influence at the article level. PLoS Biol. 14:e1002541. 10.1371/journal.pbio.1002541 27599104 PMC5012559

[B12] Kim H. J. Yoon D. Y. Kim E. S. Lee K. Bae J. S. Lee J. H. (2016). The 100 most-cited articles in neuroimaging: a bibliometric analysis. Neuroimage 139, 149–156. 10.1016/j.neuroimage.2016.06.029 27327516

[B13] Lamy C. Oppenheim C. Meder J. Mas J. (2004). Neuroimaging in posterior reversible encephalopathy syndrome. J. Neuroimaging 14, 89–96. 10.1111/j.1552-6569.2004.tb00223.x 15095552

[B14] Lariviere V. Diepeveen S. Chonaill S. N. Macaluso B. Pollitt A. Grant J. (2013). International comparative performance of mental health research, 1980–2011. Eur. Neuropsychopharmacol. 23, 1340–1347. 10.1016/j.euroneuro.2013.01.006 23452564

[B15] Larivière V. Haustein S. Mongeon P. (2015). The oligopoly of academic publishers in the digital era. PLoS ONE 10:e0127502. 10.1371/journal.pone.0127502 26061978 PMC4465327

[B16] Leydesdorff L. (1998). Theories of citation? Scientometrics 43, 5–25. 10.1007/BF02458391

[B17] Leydesdorff L. Opthof T. (2013). Citation analysis with medical subject headings (MeSH) using the web of knowledge: a new routine. J. Am. Soc. Inform. Sci. Technol. 64, 1076–1080. 10.1002/asi.22770

[B18] Leydesdorff L. Rotolo D. Rafols I. (2012). Bibliometric perspectives on medical innovation using the medical subject headings of pub med. J. Am. Soc. Inform. Sci. Technol. 63, 2239–2253. 10.1002/asi.22715

[B19] Lozano G. A. Larivière V. Gingras Y. (2012). The weakening relationship between the impact factor and papers' citations in the digital age. J. Am. Soc. Inform. Sci. Technol. 63, 2140–2145. 10.1002/asi.22731

[B20] Lundberg J. Fransson A. Brommels M. Skår J. Lundkvist I. (2006). Is it better or just the same? Article identification strategies impact bibliometric assessments. Scientometrics 66, 183–197. 10.1007/s11192-006-0013-4

[B21] Ma Y. Dong M. Zhou K. Mita C. Liu J. Wayne P. M. (2016). Publication trends in acupuncture research: a 20-year bibliometric analysis based on PubMed. PLoS ONE 11:e0168123. 10.1371/journal.pone.0168123 27973611 PMC5156436

[B22] Manca A. Martinez G. Cugusi L. Dragone D. Dvir Z. Deriu F. (2017). The surge of predatory open-access in neurosciences and neurology. Neuroscience 353, 166–173. 10.1016/j.neuroscience.2017.04.014 28433651

[B23] Miranda R. Garcia-Carpintero E. (2018). Overcitation and overrepresentation of review papers in the most cited papers. J. Informetr. 12, 1015–1030. 10.1016/j.joi.2018.08.006

[B24] Miranda R. Garcia-Carpintero E. (2019). Comparison of the share of documents and citations from different quartile journals in 25 research areas. Scientometrics 121, 479–501. 10.1007/s11192-019-03210-z

[B25] Moed H. F. (2010). Measuring contextual citation impact of scientific journals. J. Informetr. 4, 265–277. 10.1016/j.joi.2010.01.002

[B26] Moed H. F. Halevi G. (2016). On full text download and citation distributions in scientific-scholarly journals. J. Assoc. Inform. Sci. Technol. 67, 412–431. 10.1002/asi.23405

[B27] Mosa A. S. M. Yoo I. (2013). A study on PubMed search tag usage pattern: association rule mining of a full-day PubMed query log. BMC Med. Inform. Decis. Mak. 13:8. 10.1186/1472-6947-13-8 23302604 PMC3552776

[B28] Mueller S. G. Weiner M. W. Thal L. J. Petersen R. C. Jack C. R. Jagust W.. (2005). Ways toward an early diagnosis in alzheimer's disease: the alzheimer's disease neuroimaging initiative (ADNI). Alzheimer's Dement. 1, 55–66. 10.1016/j.jalz.2005.06.003 17476317 PMC1864941

[B29] Müller V. I. Cieslik E. C. Laird A. R. Fox P. T. Radua J. Mataix-Cols D.. (2018). Ten simple rules for neuroimaging meta-analysis. Neurosci. Biobehav. Rev. 84, 151–161. 10.1016/j.neubiorev.2017.11.012 29180258 PMC5918306

[B30] Poldrack R. A. Baker C. I. Durnez J. Gorgolewski K. J. Matthews P. M. Munafò M. R.. (2017). Scanning the horizon: towards transparent and reproducible neuroimaging research. Nat. Rev. Neurosci. 18, 115–126. 10.1038/nrn.2016.167 28053326 PMC6910649

[B31] Rosenkrantz A. B. Pinnamaneni N. Babb J. S. Doshi A. M. (2016). Most common publication types in radiology journals: what is the level of evidence? Acad. Radiol. 23, 628–633. 10.1016/j.acra.2016.01.002 26898526

[B32] Samartzis L. Dima D. Fusar-Poli P. Kyriakopoulos M. (2014). White matter alterations in early stages of schizophrenia: a systematic review of diffusion tensor imaging studies. J. Neuroimaging 24, 101–110. 10.1111/j.1552-6569.2012.00779.x 23317110

[B33] Schellinger P. Bryan R. Caplan L. Detre J. Edelman R. Jaigobin C.. (2010). Evidence-based guideline: the role of diffusion and perfusion MRI for the diagnosis of acute ischemic stroke: report of the therapeutics and technology assessment subcommittee of the american academy of neurology. Neurology 75, 177–185. 10.1212/WNL.0b013e3181e7c9dd 20625171 PMC2905927

[B34] Tahamtan I. Bornmann L. (2019). What do citation counts measure? An updated review of studies on citations in scientific documents published between 2006 and 2018. Scientometrics 121, 1635–1684. 10.1007/s11192-019-03243-4

[B35] Van Leeuwen T. N. Moed H. F. (2005). Characteristics of journal impact factors: the effects of uncitedness and citation distribution on the understanding of journal impact factors. Scientometrics 63, 357–371. 10.1007/s11192-005-0217-z

[B36] Wallace M. L. Larivière V. Gingras Y. (2009). Modeling a century of citation distributions. J. Informetr. 3, 296–303. 10.1016/j.joi.2009.03.010

[B37] Winkelmann R. Kim G. K. Del Rosso J. Q. (2013). Treatment of cutaneous lupus erythematosus: review and assessment of treatment benefits based on Oxford centre for evidence-based medicine criteria. J. Clin. Aesthet. Dermatol. 6, 27–38. 23320123 PMC3543290

[B38] Woo C.-W. Krishnan A. Wager T. D. (2014). Cluster-extent based thresholding in fMRI analyses: pitfalls and recommendations. Neuroimage 91, 412–419. 10.1016/j.neuroimage.2013.12.058 24412399 PMC4214144

[B39] Yeung A. W. (2018a). The 100 most cited papers concerning the insular cortex of the brain: a bibliometric analysis. Front. Hum. Neurosci. 12:337. 10.3389/fnhum.2018.00337 30210323 PMC6119810

[B40] Yeung A. W. K. (2017). Do neuroscience journals accept replications? A survey of literature. Front. Hum. Neurosci. 11:468. 10.3389/fnhum.2017.00468 28979201 PMC5611708

[B41] Yeung A. W. K. (2018b). Bibliometric study on functional magnetic resonance imaging literature (1995–2017) concerning chemosensory perception. Chemosens. Percept. 11, 42–50. 10.1007/s12078-018-9243-0

[B42] Yeung A. W. K. (2018c). An updated survey on statistical thresholding and sample size of fMRI studies. Front. Hum. Neurosci. 12:16. 10.3389/fnhum.2018.00016 29434545 PMC5790797

[B43] Yeung A. W. K. (2019a). Comparison between scopus, web of science, PubMed and publishers for mislabelled review papers. Curr. Sci. 116, 1909–1914. 10.18520/cs/v116/i11/1909-1914

[B44] Yeung A. W. K. (2019b). Higher impact factor of neuroimaging journals is associated with larger number of articles published and smaller percentage of uncited articles. Front. Hum. Neurosci. 12:523. 10.3389/fnhum.2018.00523 30687042 PMC6338050

[B45] Yeung A. W. K. Goto T. K. Leung W. K. (2017a). At the leading front of neuroscience: a bibliometric study of the 100 most-cited articles. Front. Hum. Neurosci. 11:363. 10.3389/fnhum.2017.00363 28785211 PMC5520389

[B46] Yeung A. W. K. Goto T. K. Leung W. K. (2017b). A bibliometric review of research trends in neuroimaging. Curr. Sci. 112, 725–734. 10.18520/cs/v112/i04/725-734

[B47] Yeung A. W. K. Goto T. K. Leung W. K. (2017c). The changing landscape of neuroscience research, 2006–2015: a bibliometric study. Front. Neurosci. 11:120. 10.3389/fnins.2017.00120 28377687 PMC5360093

[B48] Yeung A. W. K. Goto T. K. Leung W. K. (2018). Readability of the 100 most-cited neuroimaging papers assessed by common readability formulae. Front. Hum. Neurosci. 12:308. 10.3389/fnhum.2018.00308 30158861 PMC6104455

[B49] Yeung A. W. K. Wong N. S. M. Lau H. Eickhoff S. B. (2019). Human brain responses to gustatory and food stimuli: a meta-evaluation of neuroimaging meta-analyses. Neuroimage 202:116111. 10.1016/j.neuroimage.2019.116111 31446124

