# Supplementary figures and images for: Most Common Publication Types of Neuroimaging Literature: Papers With High Levels of Evidence Are on the Rise
Source: Front Hum Neurosci. 2020 Apr 28;14:136. doi: 10.3389/fnhum.2020.00136 (PMC7198890; doi:10.3389/fnhum.2020.00136)

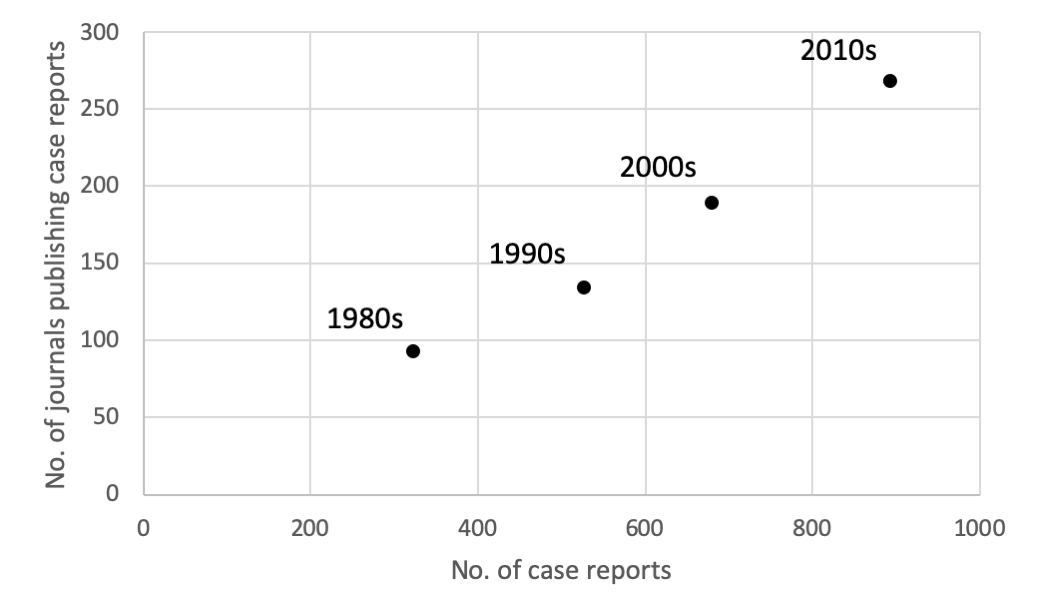

Supplement: Supplementary Figure 1 — An apparent linear relationship between the number of case reports published in each decade and the corresponding number of journals publishing them. [file Image_1.TIFF]
