# Supplementary material for: Most Common Publication Types of Neuroimaging Literature: Papers With High Levels of Evidence Are on the Rise
Source: Front Hum Neurosci. 2020 Apr 28;14:136. doi: 10.3389/fnhum.2020.00136 (PMC7198890; doi:10.3389/fnhum.2020.00136)
Supplement: Supplementary Table 1 — Year-adjusted citation counts of various publication types of neuroimaging papers, in descending order. [file Table_1.docx]

Supplementary Table 1. Year-adjusted citation counts of various publication types of neuroimaging papers, in descending order.

| Publication type | Year-adjusted citation count, mean ± SD | % of publications above the mean year-adjusted citation count | % of uncited publications |
| --- | --- | --- | --- |
| Meta-analysis | 10.0 ± 15.5 | 26.4 | 2.8 |
| Review | 5.9 ± 13.9 | 25.1 | 4.9 |
| Multicenter study | 4.7 ± 11.5 | 26.8 | 6.3 |
| Technical report | 4.5 ± 9.7 | 18.8 | 12.5 |
| Comparative study | 3.9 ± 8.5 | 26.5 | 2.5 |
| Validation study | 3.7 ± 6.4 | 27.8 | 3.9 |
| Clinical trial | 3.7 ± 5.6 | 30.4 | 2.5 |
| Guideline | 3.7 ± 4.4 | 30.0 | 0 |
| Randomized controlled trial | 3.5 ± 6.4 | 28.7 | 2.7 |
| Evaluation study | 3.0 ± 4.6 | 28.2 | 2.5 |
| Case report | 1.0 ± 2.2 | 26.1 | 11.2 |
| Editorial | 0.5 ± 1.3 | 23.5 | 42.2 |
